# Ossification Pattern of Estuarine Dolphin (*Sotalia guianensis*) Forelimbs, from the Coast of the State of Espírito Santo, Brazil

**DOI:** 10.1371/journal.pone.0127435

**Published:** 2015-05-27

**Authors:** Anna Paula Martins de Carvalho, Juliana Ywasaki Lima, Carolina Torres Azevedo, Silvina Botta, Fábio Ferreira de Queiroz, Adélia Sepúlveda Campos, Lupércio de Araújo Barbosa, Leonardo Serafim da Silveira

**Affiliations:** 1 Laboratory of Morphology and Animal Pathology, Universidade Estadual do Norte Fluminense Darcy Ribeiro, Campos dos Goytacazes, Rio de Janeiro, Brasil; 2 Laboratory of Ecology and Marine Megafauna Conservation Studies, EcoMega, College of Oceanography, Universidade Federal do Rio Grande do Sul, Rio Grande, Rio Grande do Sul, Brasil; 3 Doctor of Veterinary Medicine, Campos dos Goytacazes, Rio de Janeiro, Brasil; 4 Instituto Consciência Ambiental, Vila Velha, Espírito Santo, Brasil; New York Institute of Technology College of Osteopathic Medicine, UNITED STATES

## Abstract

The estuarine dolphin, *Sotalia guianensis*, is one of the most abundant cetacean species in Brazil. Determination of age and of aspects associated with the development of this species is significant new studies. Counts of growth layer groups in dentin are used to estimate age of these animals, though other ways to evaluate development are also adopted, like the measurement of total length (TL). This study presents a procedure to evaluate the development of the estuarine dolphin based on the ossification pattern of forelimbs. Thirty-seven estuarine dolphins found in the state of Espírito Santo, Brazil, were examined. Age was estimated, TL was measured and ossification of epiphyses was examined by radiography. We analyzed results using the Spearman correlation. Inspection of radiographs allowed evaluation of the significance of the correlation between age and development of the proximal (r = 0.9109) and distal (r = 0.9092) radial epiphyses, and of the distal ulnar epiphyses (r = 0.9055). Radiographic analysis of forelimbs proved to be an appropriate method to evaluate physical maturity, and may be a helpful tool to estimate age of these animals in ecological and population studies.

## Introduction

*Sotalia guianensis*, known as the estuarine dolphin, is one of the most abundant cetaceans in Brazil and is distributed along a large part of the South and Central American coasts [1, 2]. This species is readily observed, and therefore has been the focus of research in this geographic area, though little is known about *S*. *guianensis* by The World Conservation Union [3] and the Brazilian government [4].

The estuarine dolphin is a small cetacean of the Family Delphinidae [5]. Maximum lifespan of this species is estimated at between 30 and 35 years, though exact longevity is unknown [6]. On average, sexual maturity is reached at the age of six years, while physical maturity occurs at seven years, when the animal reaches 185 cm in total length (TL) [7]. TL is based on an axial straight-line measurement of the distance between the upper tip of the rostrum to the median notch of the tail fluke [8]. So far, no data have been published about estuarine dolphins in the state of Espírito Santo, Brazil. However, Carvalho et al. [9] report that mean TL of individuals older than seven years of age found in this state is 187.5 cm.

According to Rosas et al. [6], the stratification of a population based on developmental stages plays an important role in reproduction and population studies. The level of physical maturity reached by an individual may be assessed using several indicators, such as morphological age (assessed measuring different parts of the body, like TL), skeletal maturity (based on the development of carpals and phalanges and on the spine), sexual maturity (determined by histological evaluation of the gonads), and dental age (based on tooth formation and eruption) [10,11].

In odontocete cetaceans, age estimation is an essential parameter in studies addressing population biology and lifecycle issues [12,13]. The most common method used to estimate age is counting growth layer groups (GLGs) in tooth dentin and/or cement [12]. However, this method poses some problems, since access to teeth of these animals is difficult, and experimental procedures are far from simple and direct. TL is also often used as a parameter to estimate age [8].

In humans, one of the most common age estimate parameters is skeletal maturation, based on hand and wrist radiographs [14, 15]. In several cetacean species, like Bryde’s whale [16], the method uses forelimb radiographs as described by Ogden et al. [17], which analyzes secondary ossification centers to determine the developmental stage of animals. According to Perrin [18] and Calzada et al. [19] also, forelimbs may be used to assess physical development of individuals.

Therefore, the present study presents a method to evaluate physical maturity of *S*. *guianensis* based on the stage of skeletal development of forelimbs.

## Materials and Methods

### Ethics Statement

SISBIO (Sistema de Autorização e Informação em Biodiversidade), ICMBio (Instituto Chico Mendes de Conservação da Biodiversidade) and MMA (Ministério do Meio Ambiente) authorized the collection and use of animals for scientific purposes (S1 File).

Animals found dead at geographic coordinates between 18°35’-21°00’S and 39°43’- 40°42’ W were necropsied, and forelimbs and teeth were collected and analyzed.

This study was carried out with permission of the Sistema de Autorização e Informação em Biodiversidade—SISBIO, under permit number 20264–2.

### Animals

Thirty-seven estuarine dolphins were used in this study. These animals were found dead on the coast of the state of Espírito Santo, Brazil. Many animals presented signs of having been accidentally captured. TL and sex of each animal were determined whenever body preservation allowed. Forelimbs and teeth were collected from all carcasses for radiographic analysis and age estimation, respectively.

### Forelimb radiographs

Forelimbs of each individual were labeled, individually placed in plastic bags, and stored in a freezer. Mediolateral radiographs of pairs of forelimbs of each animal were taken at 25 mA and 75 kVp using a 30-cm ruler in a common X-ray device using a digital cassette (35 x 45 AGFA) and developed in a digital radiography device (AGFA). Measurements were made using the software eFilmLite, which also digitally retouched digital radiographs to improve definition of the epiphyses analyzed.

Radiographs of forelimbs were individually examined and classified into stages according to the secondary ossification centers of the proximal epiphyses of metacarpals. Metacarpal bones were not categorized, since they did not present a fusion pattern associated with *S*. *guianensis* development [20]. The region of interest spanned from the distal humerus to the metacarpus II, according to the classification system proposed by Ogden et al. [17]:

*Stage 0*: No secondary ossification center is observed.

*Stage 1*: Secondary ossification center is visible, but accounts for less than 50% of the latitudinal width of the adjacent metaphysis, that is, it corresponds to less than 50% of the width of the bone epiphysis.

*Stage 2*: Secondary ossification center is well established, and ranges from 50% to the full width of the metaphysis. Physis is evident as a distinct line between the secondary center and the metaphysis.

*Stage 3*: There is thinning of the radiolucent physis, with formation of more dense bone between the metaphysis and the secondary ossification center.

*Stage 4*: Closure of the physis is evident, with the formation of trabecular osseous bridges between metaphysis and the secondary ossification center. This stage is marked by variation, but beginning closure is the only criterion to be confirmed.

*Stage 5*: Closure of the physis is complete, with a more radiopaque line traversing the whole bone width.

*Stage 6*: Remodeling starts with the disappearance of the radiopaque line present in stage 5.

### Age estimates

Teeth were processed according to Hohn et al. [21]. Briefly, teeth were cut in slices using a low-speed diamond metallographic saw to obtain the central portion, with the aim of using the longest portion of teeth. Afterward, each central portion was decalcified with a commercial decalcifying acid (RDO) and cut into 25-μm sections using a freezing microtome. Sections were then stained in Mayer’s haematoxylin for 30 minutes. An ammonia solution was used to enhance staining. Sections were mounted on slides in 100% glycerin, and inspected using a magnifying glass and a microscope (40x) to count GLGs in dentin according to Perrin & Myrick [22].

**Fig 1:**
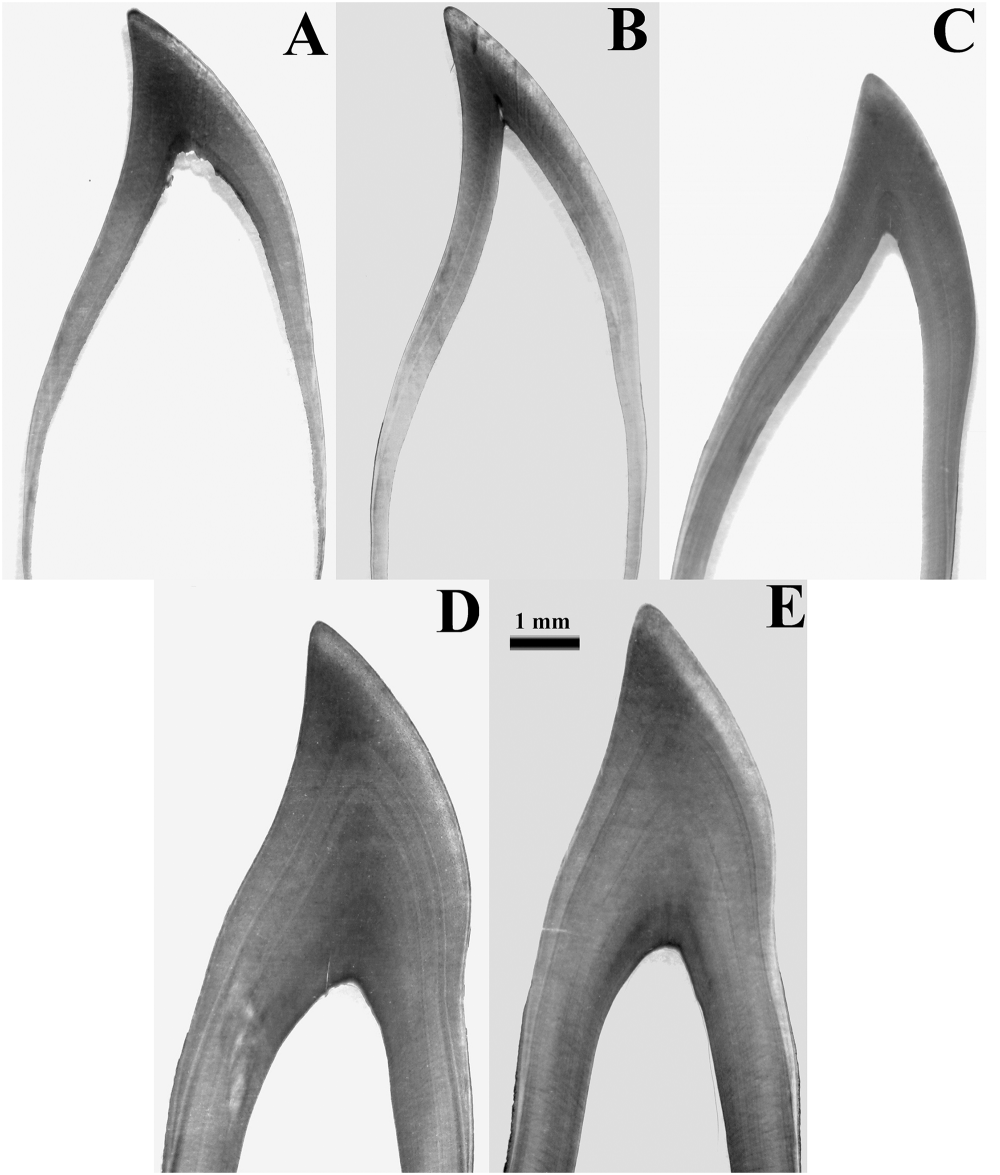
Age determination by growth layer groups in *Sotalia guianensis*. (A) newborn, (B) 0.5 year, (C) 1 year, (D) and (E) 5 years. Microscopy of tooth (40x magnification) reveals growth layers.

Each GLG (formed by a stained band and an unstained band) was considered to represent one year of age [21]. Animals with only one neonatal line (unstained layer that signals birth) were considered ½ year old, while those that did not present the neonatal line were considered newborns and given zero year of age (Fig 1).

### Statistical analysis

All parameters were submitted to the Mann-Whitney U test to assess gender differences. Relationship between age and development stage of each epiphysis of the forelimbs was evaluated using the Spearman correlation (*r*). Linear and nonlinear regression models were adjusted to the data of variables that presented significant correlation. All statistical tests were carried out at 95% significance level. Non-parametric tests were used due to the non-uniformity of data obtained in the Shapiro-Wilk test (p > 0.05). Analyses were completed using the Past 1.91 software, and curves were plotted using the Curve Expert 1.4 software.

## Results

Of the 37 estuarine dolphins analyzed, eight were females, 24 were males, and five could not have their sex determined. No statistically significant differences were observed between males and females in any of the parameters assessed (TL and degree of ossification of each region of forelimb epiphyses) in the Mann-Whitney U test (p > 0.05). Age of animals varied between zero and 20 years, while TL ranged from 92 cm to 194 cm (Table 1). No differences were observed between left and right forelimbs in ossification degree or in development times, and epiphyses were observed in all degrees of ossification described (Table 2).

**Table 1:** Minimum and maximum TL, standard deviation (SD), and age of animals analyzed.

Parameters	Minimum	Mean	Maximim	SD
**TL**	92	161.95	194	27.02
**Age**	0	5.01	20	5.87

**Table 2:** Data evaluated in this study.

Animal	Humerus	Proximal Radius	Proximal Ulna	Distal Radius	Distal Ulna	Metacarpus	Age	Sex	TL (cm)
1	3	2	2	1	1	1	Newborn	M	96
2	3	3	3	1	0	0	Newborn	M	92
3	3	3	3	2	2	1	Newborn	U	122
4	4	*3*	3	2	1	2	<1	M	-
5	3	3	3	2	2	2	<1	F	-
6	4	3	3	2	2	0	<1	M	142
7	4	3	3	2	2	2	1	M	144.5
8	4	4	4	2	2	2	1	U	149
9	4	3	3	2	2	2	1	M	129
10	4	3	3	2	2	2	1	M	140
11	4	4	4	3	3	2	2	F	151.5
12	4	3	3	2	2	2	2	M	190
13	4	4	6	3	3	1	2	M	172
14	4	4	4	2	2	1	2	F	134
15	6	4	6	3	3	3	2	M	165
16	6	4	6	3	3	2	2	M	153
17	4	4	4	2	2	2	2	M	152
18	4	4	4	3	3	2	3	F	169
19	6	5	6	4	4	2	3	M	-
20	6	6	6	3	3	2	3	M	187
21	6	6	6	4	5	2	3	M	164
22	4	4	4	2	2	2	4	M	151.5
23	6	6	6	3	4	2	4	U	-
24	6	6	6	4	4	2	5	F	185
25	6	6	6	4	4	3	5	M	180
26	6	6	6	4	4	3	5	M	-
27	6	6	6	4	5	4	5	M	184.5
28	6	6	6	5	5	4	5	U	174
29	6	6	6	4	5	4	6	M	181
30	6	6	6	4	4	2	6	M	161.3
31	6	6	6	5	5	2	8	M	190
32	6	6	6	4	6	4	8	M	187
33	6	6	6	6	6	6	16	F	183
34	6	6	6	6	6	6	17	U	194
35	6	6	6	6	6	6	20	F	192.5
36	6	6	6	6	6	4	20	F	183
37	6	6	6	4	4	3	20	M	183.5

The number attributed to each bone indicates the ossification stage of the epiphysis (1–6). Animals under 1 year of age are those that presented the neonatal line, but not the 1-year line. When total length (TL) is not given, it is because animals found were at an advanced decay stage. M: male; F: female; U: undetermined.

However, significant correlation was observed between all epiphyses and dental age (*r* > 0.7, df = 37, p < 0.05). The highest correlations with dental age were for proximal (r = 0.9109) and distal (r = 0.9092) radial epiphyses, and for distal ulnar epiphyses (r = 0.9055). The other correlations were humerus (r = 0.8353), proximal ulna (r = 0.8262), and metacarpus (r = 0.7821).

Ossification of forelimbs followed the proximal-distal direction. It started with the distal humerus and the proximal radius and ulna, followed by distal radius and ulna ossification, followed by the ossification of the metacarpus.

**Fig 2:**
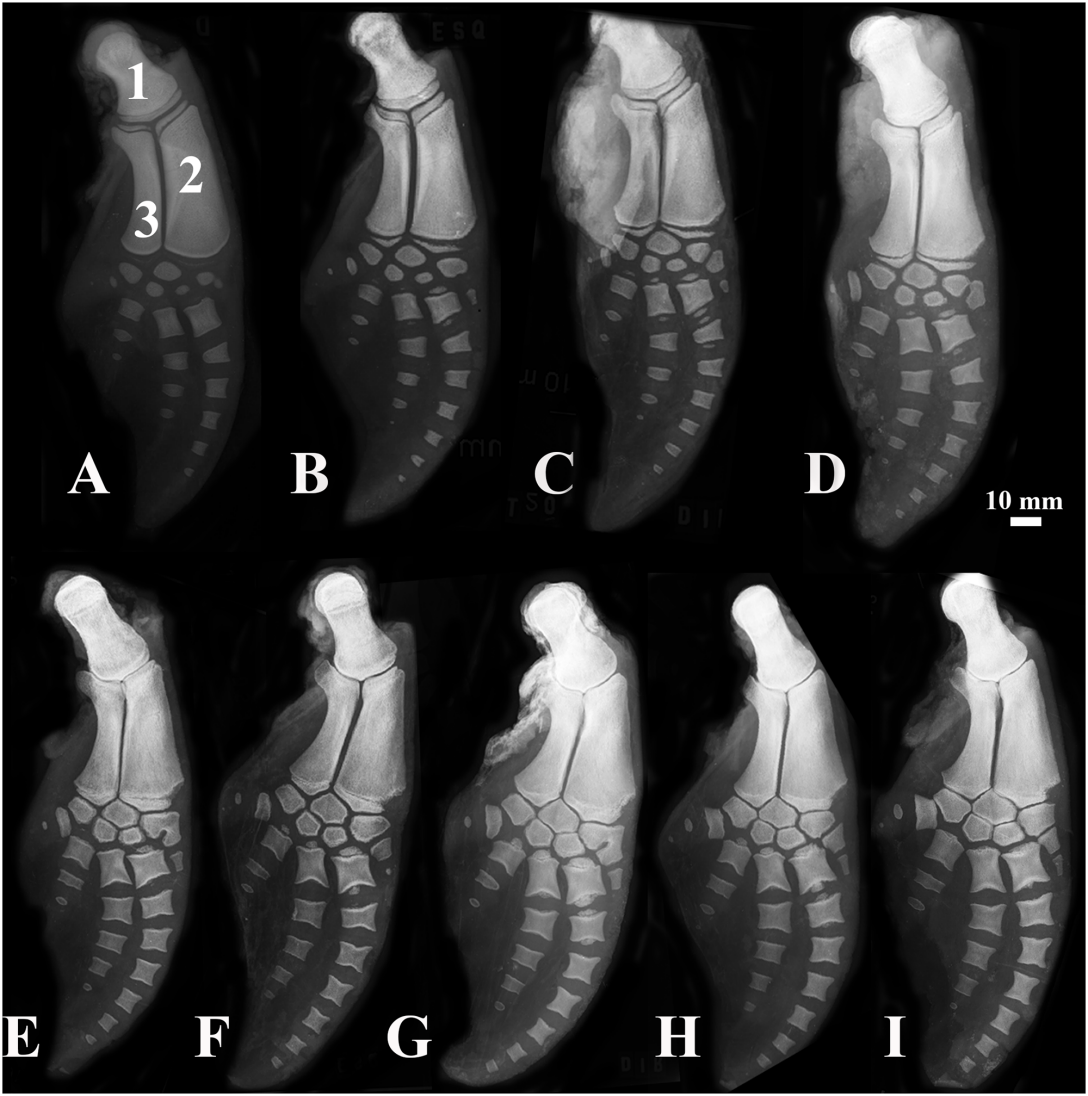
Forelimbs of the estuarine dolphin, *Sotalia guianensis*. (1) Humerus, (2) Radius, (3) Ulna. (A) Newborn, (B) animal under 1 year old, (C) 1-year-old, (D) 2-year-old, (E) 3-year-old, (F) 4-year-old, (G) 5-year-old, (H) 6-year-old, and (I) 8-year-old animals.

Epiphyseal ossification increased with age, especially the ossification of ulnar and radial distal epiphyses, which started at stage 0 in newborns and increased to stage 6 in mature animals, which is shown by the loss of the radiopaque line traversing the whole bone width (Fig 2).

Considering the humerus, ulnar and radial proximal epiphyses, animals at lower stages were also the youngest. Radial and ulnar proximal epiphyses at stage 2 were observed in newborns. Animals that presented incipient ossification in distal radial, ulnar and metacarpal epiphyses were the younger ones, with stages later than 4.

Animals with ulnar and radial distal epiphyses at stage 4 had TL greater than 160 cm, were over three years old, and presented advanced ossification of vertebral epiphyses.

## Discussion

No differences were observed in ossification stage or in development time between left and right forelimbs of one animal. These results match ossification stage and development in *Delphinus delphis* [23] and *Stenella coeruleoalba* [24]. However, Ogden et al. [17] described differences between left and right forelimbs of *Phocoenoides dalli* and *Globicephala macrorhyncus*.

Differences between males and females were reported for *Phocoena sinus* [Mellor et al.] and *Phocoena phocoena* [25].

Calzada et al. [19] observed that *Stenella coeruleoalba* fetuses and newborns have radius and proximal ulna at stage 3, with closure of the physis starting when these animals are two years old and finishing when they are three. In the present study, the animals examined presented a similar ossification stage, with some newborns showing stage 2 epiphyses.

Ogden et al. [16] and Stockin et al. [17] agree that stage 4 in ulnar and radial distal epiphyses indicates sexual maturity. A previous study established six years as the sexual maturity age of estuarine dolphins on the northern coast of the state of Rio de Janeiro, Brazil [7]. Results of that investigation showed that distal radial and ulnar epiphyses of all animals over six years of age were at least stage 4, though not every individual at this ossification stage was that age. It follows either that the estuarine dolphins in the state of Espírito Santo, Brazil reach sexual maturity at an early age, or that this relationship is not valid for this species, since some 4-year-old dolphins were at stage 4 of ossification. Therefore, sexual maturity of the animals examined in the present study might shed more light on the relationship between this parameter and ossification degree, as proposed by the authors cited.

Calzada et al. [19] reported that the distal humerus of *Stenella coeruleoalba* is at stages 3 and 4 of ossification in fetuses and newborns, and at stages 5 or 6 in older animals. The results obtained in the present study indicate that ossification patterns may vary across delphinids, since some young animals presented stage 4 ossification, while some animals of 3 or 4 years of age also were at this stage.

The progression of epiphysis ossification observed is identical to that reported by Galatius [25] for *Phocoena phocoena*. The process starts with the humerus, proceeds with the radial and ulnar proximal epiphyses and then with the radial and ulnar distal epiphyses, almost concomitantly with the ossification of the metacarpus. However, in the present study only metacarpal II was analyzed, while Galatius et al. [25] correlated the development of epiphyses to metacarpal IV.

This demonstrates the significant correlation between age and radial and ulnar distal epiphyses, as reported by Calzada et al. [19] and Fragoso [26]. However, in the present study, the correlation between age and radial proximal epiphyses is stronger than the correlations between age and distal radial and ulnar epiphyses, though these were statistically significant. This suggests that all three epiphyses should be evaluated in order to obtain a more accurate correlation between ossification and age.

Mellor et al. [27] studied older, physically mature *Phocoena sinus* individuals, whose humeral, ulnar and metacarpal ossification was complete. Ossification was unfinished only in the distal radial epiphysis of 11-year-old females. These results are similar to the findings observed in the present study, where all animals considered physically mature were those whose epiphyseal development was at this stage. Nevertheless, we could not compare the progression of the ossification of *S*. *guianensis* with that of *Phocoena*, since Mellor et al. [27] only studied mature animals.

For Butti et al. [28], radiography is useful to investigate bone development, but not age. Although the technique does not permit accurate estimation of age, the high correlation between age and ossification of epiphyses in forelimbs of estuarine dolphins allowed determining the developmental stage of the animals analyzed.

Analysis of forelimbs is not widely used as a method to establish age of cetaceans, but the results obtained in this study prove its higher efficiency in determining skeletal maturity, compared with other traditional methods, like TL.

Radiography is a less invasive, less complex, faster and cheaper method, compared to age estimation based on GLGs in dentin. Additionally, it can be performed on live animals, even in the field using portable X-ray devices, which do not require long exposure times. The removal of one tooth demands anesthesia, while radiographs may be conducted while merely sedating animals, which is less deleterious to them. In addition, radiography avoids the stress associated with tooth extraction, which may expose animals to pathogens.

## Conclusions

Radiography of forelimbs of the estuarine dolphin may be used to evaluate physical development and estimate the age of these animals. Also, the technique is easy to carry out, less invasive than other traditional methods, and therefore can play an important part in the evaluation of populations and of new parameters for this species.

## Supporting Information

S1 File Permission for animal use. (PDF)

## References

[1] Simões-Lopes PC. Ocorrência de uma população de *Sotalia fluviatilis* (Gervais, 1853) (Cetacea, Delphinidae) no limite sul de sua distribuição, Santa Catarina, Brasil. Biotemas 1988; 1: 57–62.

[2] Flores PAC, Silva VMF. Tucuxi and Guiana Dolphin—*Sotalia fluviatilis* and *S*. *guianensis* In: Perrin WF, Würsig B, Thewissen JGM, editors Encyclopedia of marine mammals, 2nd ed Amsterdam: Academic Press; 2008 pp. 1188–1192.

[3] IUCN. Lista Vermelha de Espécies Ameaçadas Versão 2012/2. Available: http://www.iucnredlist.org. Accessed 14 January 2015.

[4] IBAMA. Mamíferos aquáticos do Brasil: plano de ação. Versão II Brasília: Edições IBAMA; 2001.

[5] Fettuccia DC. Comparação osteológica nas espécies do gênero *Sotalia* Gray, 1866 no Brasil (Cetacea, Delphinidae). M.Sc. Dissertation, Universidade Federal do Aamazonas. 2006. Available: http://obraslivres.com/obras/63116/comparacao-osteologica-nas-especies-do-genero-sotalia-gray-1866-no-brasil-cetacea-delphinidae

[6] Rosas FCW, Barreto AS, Monteiro-Filho ELA. Age and growth of the estuarine dolphin (*Sotalia guianensis*) (Cetacea, Delphinidae) on the Paraná coast, southern Brazil. Fish Bull. 2003; 101: 377–383.

[7] Ramos RMA, Di Benedito APM, Lima NRW. Growth parameters of *Pontoporia blainvillei* and *Sotalia fluviatilis* (Cetacea) in northern Rio de Janeiro, Brazil. Aquat Mamm. 2000; 26: 65–75. 10.1590/s0034-71082000000200012 10959112

[8] Norris KS. Standardized methods for measuring and recording data on the smaller cetaceans. J Mammal. 1961; 42: 471–476.

[9] Carvalho APM, Ywasaki J, Azevedo CT, Campos AS, Queiroz FF, Pontes LAE, et al Crescimento e desenvolvimento de boto-cinza (*Sotalia guianensis*) do litoral do Espírito Santo. Arq Bras Med Vet Zootec. 2012; 64: 205–208.

[10] Martins RJC, Sakima T. Considerações sobre a previsão do surto de crescimento puberal. Ortod. 1977; 10: 164–170. 275777

[11] Moscatiello VAM, Lederman H, Moscatiello RA, Faltin Júnior K, Moscatiello RM. Maturação das vértebras cervicais e sua correlação com a idade óssea da mão e punho como indicadores no tratamento ortodôntico. R Dental Press. Ortodon Ortop Facial. 2008; 13: 92–100.

[12] Hohn AA. Reading between the lines: analysis of age determination in dolphins In: Leatherwood S, Reeves RR, editors. San Diego: The Bottlenose Dolphin. Academic Press; 1990 pp. 575–585.

[13] Sydney NV, Monteiro-Filho ELA. Efficiency of wear and decalcification technique for estimating the age of estuarine dolphin *Sotalia guianensis* . J Biosci. 2011; 36: 117–121. 21451253 10.1007/s12038-011-9005-5

[14] Tavano O, Freitas JAS, Lopes ES. Greulich & Pyle e Tanner & Whitehouse: comparação entre duas tabelas de avaliação de idade biológica através do desenvolvimento ósseo. Pediatr Clín. 1982; 6: 7–21.

[15] Santos SCBN, Almeida RR. Estudo comparativo de dois métodos de avaliação da idade esquelética utilizando tele radiografias em norma lateral e radiografias carpais. Ortod. 1999; 32: 33–45. 10878884

[16] Stockin KA, Wiseman N, Hartman A, Moffat N, Roe WD. Use of radiography to determine age class and assist with the post-mortem diagnostics of a Bryde’s whale (*Balaenoptera brydei)* . NZ J Mar Freshw Res. 2008; 42: 307–313.

[17] Ogden JA, Conlogue GJ, Rhodin AG. Roentgenographic indicators of skeletal maturity in marine mammals (Cetacea). Skelet Radiol. 1981; 7: 119–123. 10.1007/BF00347376 7330661

[18] Perrin WF. Variation of spotted and spinner porpoise (genus *Stenella*) in the eastern tropical Pacific and Hawaii. Bull. Scripps Inst Oceanogr. 1975; 21: 1–206.

[19] Calzada N, Aguilar A, Lockyer C, Grau E. Patterns of growth and physical maturity in the western Mediterranean striped dolphin, *Stenella coeruleoalba* (Cetacea: Odontoceti). Can J Zool. 1997; 75: 632–637.

[20] Menezes ME, Simões-Lopes PC. Osteologia e morfologia da aleta peitoral da forma marinha de *Sotalia fluviatilis* (Cetacea-Delphinidae) no litoral do sul do Brasil. Estud Biol. 1996; 4:. 23–31. doi: 10.1002/(SICI)1097-0193(1996)4:1&lt;23::AID-HBM2&gt;3.0.CO;2-R 20408184

[21] Hohn A, Scott MD, Wells RS, Sweeney JC, Irvine AB. Growth layers in teeth from known-age free-rangin*g* bottlenose dolphins. Mar Mamm Sci.1989; 5: 315–342.

[22] Perrin WF, Myrick AC Age determination of toothed whales and sirenians Reports of the International Whaling Commission, Special Issue 3 Cambridge, UK; 1980.

[23] Hui CA. Correlates of maturity in the common dolphin, *Delphinus delphis* . Fish Bull. 1979; 77: 295–300.

[24] Di Giancamillo M, Rattegni G, Podest M, Cagnolaro L, Cozzi B, Leonardi L. Postnatal ossification of the thoracic limb in striped dolphins (*Stenella coeruleoalba*) (Meyen, 1833) from the Mediterranean Sea. Can J Zool. 1998; 76: 1286–1293.

[25] Galatius A, Andersen, MER, Haugan B, Langhoff HE, Jespersen Å. Timing of epiphyseal development in the flipper skeleton of the harbor porpoise (*Phocoena phocoena*) as an indicator of paedomorphosis. Acta Zool. 2006; 87: 77–82

[26] Fragoso ABL. Alterações morfológicas e patológicas em esqueletos e nadadeiras peitorais de boto-cinza, *Sotalia guianensis* (Van Beneden, 1864) do litoral brasileiro. PhD Thesis, Universidade Federal do Rio de Janeiro. 2006.

[27] Mellor L, Cooper LN, Torre J, Brownell RL Jr.. Paedomorphic ossification in porpoises with an emphasis on the vaquita (Phocoena sinus). Publications, Agencies and Staff of the U.S. Department of Commerce; 2009.

[28] Butti C, Corain L, Cozzi B, Podestà M, Pirone A, Affronte M, Zotti A. Age estimation in the Mediterranean bottlenose dolphin *Tursiops truncatus* (Montagu 1821) by bone density of the thoracic limb. J Anat. 2007; 211: 639–646. 17850286 10.1111/j.1469-7580.2007.00805.x PMC2375788

