# Supplementary material for: Ossification Pattern of Estuarine Dolphin (Sotalia guianensis) Forelimbs, from the Coast of the State of Espírito Santo, Brazil
Source: PLoS One. 2015 May 27;10(5):e0127435. doi: 10.1371/journal.pone.0127435 (PMC4446342; doi:10.1371/journal.pone.0127435)
Supplement: S1 File — (PDF) [file pone.0127435.s001.pdf]

Autorização para atividades com finalidade científica

Número: 20264-2	Data da Emissão: 21/06/2011 09:11

Dados do titular

Nome: Lupércio Araújo Barbosa	CPF: 621.448.397-00
Título do Projeto: MONITOTAMENTO DE ENCALHE DE CETÁCEOS E PINIPEDES NO LITORAL DO ESPIRITO SANTO	
Nome da Instituição : Organização Consciência Ambiental - ORCA	CNPJ: 39.830.211/0001-97

SISBIO

Cronograma de atividades

#	Descrição da atividade	Início (mês/ano)	Fim (mês/ano)
1	Monitoramento de cetáceos no litoral do ES	05/2011	05/2013
2	MONITOTAMENTO DE ENCALHE DE CETÁCEOS E PINIPEDES NO LITORAL DO ESPIRITO SANTO	05/2011	05/2013

De acordo com o art. 33 da IN 154/2009, esta autorização tem prazo de validade equivalente ao previsto no cronograma de atividades do projeto.

Observações e ressalvas

1	As atividades de campo exercidas por pessoa natural ou jurídica estrangeira, em todo o território nacional, que impliquem o deslocamento de recursos humanos e materiais, tendo por objeto coletar dados, materiais, espécimes biológicos e minerais, peças integrantes da cultura nativa e cultura popular, presente e passa da, obtidos por meio de recursos e técnicas que se destinem ao estudo, à difusão ou à pesquisa, estão sujeitas a autorização do Ministério de Ciência e Tecnologia.
2	Esta autorização NÃO exime o pesquisador titular e os membros de sua equipe da necessidade de obter as anuências previstas em outros instrumentos legais, bem como do consentimento do responsável pela área, pública ou privada, onde será realizada a atividade, inclusive do órgão gestor da unidade de conservação estadual, distrital ou municipal, ou do proprietário, arrendatário, posseiro ou morador de área dentro dos limites de unidade de conservação federal cujo processo de regularização fundiária encontra-se em curso.
3	Este documento somente poderá ser utilizado para os fins previstos na Instrução Normativa IBAMA n° 154/2007 ou na Instrução Normativa ICMBio n° 10/2010, no que especifica esta Autorização, não podendo ser utilizado para fins comerciais, industriais ou esportivos. O material biológico coletado deverá ser utilizado para atividades científicas ou didáticas no âmbito do ensino superior.
4	A autorização para envio ao exterior de material biológico não consignado deverá ser requerida por meio do endereço eletrônico www.ibama.gov.br (Serviços on-line - Licença para importação ou exportação de flora e fauna - CITES e não CITES). Em caso de material consignado, consulte www.icmbio.gov.br/sisbio - menu Exportação.
5	O titular de licença ou autorização e os membros da sua equipe deverão optar por métodos de coleta e instrumentos de captura direcionados, sempre que possível, ao grupo taxonômico de interesse, evitando a morte ou dano significativo a outros grupos; e empregar esforço de coleta ou captura que não comprometa a viabilidade de populações do grupo taxonômico de interesse em condição in situ.
6	Este documento não dispensa o cumprimento da legislação que dispõe sobre acesso a componente do patrimônio genético existente no território nacional, na plataforma continental e na zona econômica exclusiva, ou ao conhecimento tradicional associado ao patrimônio genético, para fins de pesquisa científica, bioprospecção e desenvolvimento tecnológico. Veja maiores informações em www.mma.gov.br/cgen.
7	Em caso de pesquisa em UNIDADE DE CONSERVAÇÃO, o pesquisador titular desta autorização deverá contactar a administração da unidade a fim de CONFIRMAR AS DATAS das expedições, as condições para realização das coletas e de uso da infra-estrutura da unidade.
8	As atividades contempladas nesta autorização NÃO abrangem espécies brasileiras constante de listas oficiais (de abrangência nacional, estadual ou municipal) de espécies ameaçadas de extinção, sobreexplotadas ou ameaçadas de sobreexplotação.

Outras ressalvas

1	Capturar animais vivos in situ só qdo encalhados.Só encaminhar animais p/ o centro de reabilitação qdo não estiverem em boas condições físicas. Seguir a Recomendação do SCAR.Pinípedes antárticos/subantárticos encaminhados p/ centros de reabilitação não sejam liberados no ambiente natural(Arctocephalus gazella,Arctocephalus tropicalis,Mirounga leonina,Lobodon carcinophaga,Hydrurga leptonyx,Leptonychotes weddelli).Destino final dos pinípedes reabilitados tenha anuência do CMA/ICMBio. Atender INs.

Equipe

#	Nome	Função	CPF	Doc. Identidade	Nacionalidade
1	Lorena Garioli Santos	Bióloga	109.486.997-01	1885406 SPTC-ES	Brasileira
2	Leonardo Serafim da Silveira	Médico Veterinário	007.013.307-75	4921 CRMV-RJ	Brasileira
3	Alexandre D'Ávila Charpinel	Biólogo	124.374.227-57	2150989 SPTC-ES	Brasileira

Locais onde as atividades de campo serão executadas

#	Município	UF	Descrição do local	Tipo
1		ES	Vila Velha	Fora de UC
2		ES	Guarapari	Fora de UC

Este documento (Autorização para atividades com finalidade científica) foi expedido com base na Instrução Normativa nº154/2007. Através do código de autenticação abaixo, qualquer cidadão poderá verificar a autenticidade ou regularidade deste documento, por meio da página do Sisbio/ICMBio na Internet (www.icmbio.gov.br/sisbio).

SISBIO

Código de autenticação: 14137363

Autorização para atividades com finalidade científica

Número: 20264-2	Data da Emissão: 21/06/2011 09:11

Dados do titular

SISBIO

Nome: Lupércio Araújo Barbosa	CPF: 621.448.397-00
Título do Projeto: MONITOTAMENTO DE ENCALHE DE CETÁCEOS E PINIPEDES NO LITORAL DO ESPIRITO SANTO	
Nome da Instituição : Organização Consciência Ambiental - ORCA	CNPJ: 39.830.211/0001-97

Atividades X Táxons

#	Atividade	Táxons
1	Coleta/transporte de amostras biológicas in situ	Phocidae, Cetacea, Otariidae
2	Coleta/transporte de espécimes da fauna silvestre in situ	Phocidae (*Qtde: 10), Cetacea (*Qtde: 30), Otariidae (*Qtde: 10)
3	Manutenção temporária (até 24 meses) de vertebrados silvestres em cativeiro	Phocidae, Otariidae, Cetacea

* Qtde. de indivíduos por espécie/localidade/unidade de conservação, a serem coletados durante um ano.

Material e métodos

1	Amostras biológicas (Mamíferos Aquáticos:cetáceos, sirênios e pinípedes)	Fragmento de tecido/órgão, Animal morto ou partes (carcaça)/osso/pele, Ectoparasita, Fezes, Regurgitação/conteúdo estomacal, Sangue
2	Método de captura/coleta (Mamíferos Aquáticos:cetáceos, sirênios e pinípedes)	Captura manual

Destino do material biológico coletado

#	**Nome local destino**	**Tipo Destino**
1	Organização Consciência Ambiental - ORCA	Instituições de pesquisas e Universidades

Este documento (Autorização para atividades com finalidade científica) foi expedido com base na Instrução Normativa nº154/2007. Através do código de autenticação abaixo, qualquer cidadão poderá verificar a autenticidade ou regularidade deste documento, por meio da página do Sisbio/ICMBio na Internet (www.icmbio.gov.br/sisbio).

SISBIO

Código de autenticação: 14137363

Ministério do Meio Ambiente - MMA
Instituto Chico Mendes de Conservação da Biodiversidade - ICMBio
Sistema de Autorização e Informação em Biodiversidade - SISBIO

Autorização para atividades com finalidade científica

Número: 20264-2	Data da Emissão: 21/06/2011 09:11

Dados do titular

Nome: Lupércio Araújo Barbosa	CPF: 621.448.397-00
Título do Projeto: MONITOTAMENTO DE ENCALHE DE CETÁCEOS E PINIPEDES NO LITORAL DO ESPIRITO SANTO	
Nome da Instituição : Organização Consciência Ambiental - ORCA	CNPJ: 39.830.211/0001-97

SISBIO

Registro de coleta imprevista de material biológico

De acordo com a Instrução Normativa nº154/2007, a coleta imprevista de material biológico ou de substrato não contemplado na autorização ou na licença permanente deverá ser anotada na mesma, em campo específico, por ocasião da coleta, devendo esta coleta imprevista ser comunicada por meio do relatório de atividades. O transporte do material biológico ou do substrato deverá ser acompanhado da autorização ou da licença permanente com a devida anotação. O material biológico coletado de forma imprevista, deverá ser destinado à instituição científica e, depositado, preferencialmente, em coleção biológica científica registrada no Cadastro Nacional de Coleções Biológicas (CCBIO).

Táxon*	Qtde.	Tipo de amostra	Qtde.	Data

* Identificar o espécime no nível taxonômico possível.

Este documento (Autorização para atividades com finalidade científica) foi expedido com base na Instrução Normativa nº154/2007. Através do código de autenticação abaixo, qualquer cidadão poderá verificar a autenticidade ou regularidade deste documento, por meio da página do Sisbio/ICMBio na Internet (www.icmbio.gov.br/sisbio).

SISBIO

Código de autenticação: 14137363
